# Factors of acute respiratory infection among under-five children across sub-Saharan African countries using machine learning approaches

**DOI:** 10.1038/s41598-024-65620-1

**Published:** 2024-07-09

**Authors:** Haile Mekonnen Fenta, Temesgen T. Zewotir, Saloshni Naidoo, Rajen N. Naidoo, Henry Mwambi

**Affiliations:** 1 Discipline of Public Health Medicine, School of Nursing and Public Health College of Health Sciences, University of KwaZulu-Natal, Durban, South Africa; 2 Department of Statistics, College of Science, Bahir Dar University, Bahir Dar, Ethiopia; 3 School of Mathematics, Statistics and Computer Science, College of Agriculture Engineering and Science, University of KwaZulu-Natal, Durban, South Africa; 4 Discipline of Occupational and Environmental Health, School of Nursing and Public Health, College of Health Sciences, University of KwaZulu-Natal, Durban, South Africa

**Keywords:** Machine learning, Acute respiratory infection, Demographic health survey, Feature selection, Ecology, Diseases, Health care, Medical research, Risk factors, Mathematics and computing

## Abstract

Symptoms of Acute Respiratory infections (ARIs) among under-five children are a global health challenge. We aimed to train and evaluate ten machine learning (ML) classification approaches in predicting symptoms of ARIs reported by mothers among children younger than 5 years in sub-Saharan African (sSA) countries. We used the most recent (2012–2022) nationally representative Demographic and Health Surveys data of 33 sSA countries. The air pollution covariates such as global annual surface particulate matter (PM 2.5) and the nitrogen dioxide available in the form of raster images were obtained from the National Aeronautics and Space Administration (NASA). The MLA was used for predicting the symptoms of ARIs among under-five children. We randomly split the dataset into two, 80% was used to train the model, and the remaining 20% was used to test the trained model. Model performance was evaluated using sensitivity, specificity, accuracy, and the area under the receiver operating characteristic curve. A total of 327,507 under-five children were included in the study. About 7.10, 4.19, 20.61, and 21.02% of children reported symptoms of ARI, Severe ARI, cough, and fever in the 2 weeks preceding the survey years respectively. The prevalence of ARI was highest in Mozambique (15.3%), Uganda (15.05%), Togo (14.27%), and Namibia (13.65%,), whereas Uganda (40.10%), Burundi (38.18%), Zimbabwe (36.95%), and Namibia (31.2%) had the highest prevalence of cough. The results of the random forest plot revealed that spatial locations (longitude, latitude), particulate matter, land surface temperature, nitrogen dioxide, and the number of cattle in the houses are the most important features in predicting the diagnosis of symptoms of ARIs among under-five children in sSA. The RF algorithm was selected as the best ML model (AUC = 0.77, Accuracy = 0.72) to predict the symptoms of ARIs among children under five. The MLA performed well in predicting the symptoms of ARIs and associated predictors among under-five children across the sSA countries. Random forest MLA was identified as the best classifier to be employed for the prediction of the symptoms of ARI among under-five children.

## Introduction

Acute Respiratory Infections (ARIs) are among the most common childhood illnesses which accounts for more than 6% of the global disease burden. ARIs are the leading cause of death among children under the age of five^1,2^. Worldwide, ARIs caused 16% of all deaths in 2015 and killed nearly one million children under the age of five, which is greater than the burden of diarrheal illness and malaria combined^2–4^. According to the World Health Organization (WHO) in 2019, in African and European regions, the under-five death rate due to ARIs was 73/1000 and 9/1000 live births respectively^1,5^, i.e. the African region under-five death rate was almost eight times higher than the European region. Different literature reported that symptoms of ARIs in under-5-year-old children are directly related to the population’s environmental, socioeconomic, and cultural variables^2,6–10^. Moreover, air pollution disproportionately affects the under-five children residing in low and middle-income countries (LMICs), including sSA. More than 89% of deaths due to air pollution occurred in LMICs, mainly in Africa and Asia^11^. Africa accounts for the highest excess mortality from ambient air pollution among under-five children, to which ARIs were suggested as a potential contributor^11,12^. It is confirmed that 92% of the world's population lives in areas where the air quality index (AQI) limit is exceeded (> 100, AQI near 100 is usually considered safe)^13^ and about 4.2 million people die every year from many diseases due to air pollution. Under-five children are at greater risk than the other population groups from many of the adverse health effects of air pollution, mainly due to a combination of physiological, environmental, and behavioral factors. Besides, children spend most of their time outside engaging in physical activities and playing, they breathe air located closer to the ground, where some of the air pollutants are at a higher concentration, and they have a higher breathing rate than adults increasing their risk of exposure^14–16^.

Previous studies attempted to identify the determinant factors of ARIs among under-five children^2,6–12^ using linear and non-linear regression models. As far as the researcher’s knowledge is concerned, there exist a few previous studies^17–20^ that applied machine learning algorithms to predict the ARIs among under-five children using air pollution factors. So far, these machine learning algorithms have not been extensively applied to the available cross-sectional datasets in low- and middle-income countries (LMICs). Hence, we applied machine learning (ML) algorithms to investigate the effects of air pollutants (such as Particulate Matter (PM2.5), nitrogen dioxide (NO_2_)), climate factors (temperature, land surface temperature, wet day), health-related information, and socio-demographic factors. Furthermore, a generic prediction framework is lacking for reliable assessment of the symptoms of respiratory infections among children under 5 years using a large-scale dataset employing MLA. To the best of our knowledge, this is the first study that employed different ML techniques to select and identify the associated risk factors with symptoms of ARIs in sSA countries. This MLA approach places the features according to their importance considers the selected risk factors (features) simultaneously in an unbiased manner and identifies the pattern of information, which is crucial to make a prediction. The objective of this study was twofold: first, to reveal the possible features for determining the ARIs among children, and second, to explore machine learning algorithms by considering the best possible features for predicting the ARIs among children in sub-Saharan African countries.

## Materials and methods

### Data sources and variables

**Figure 1:**
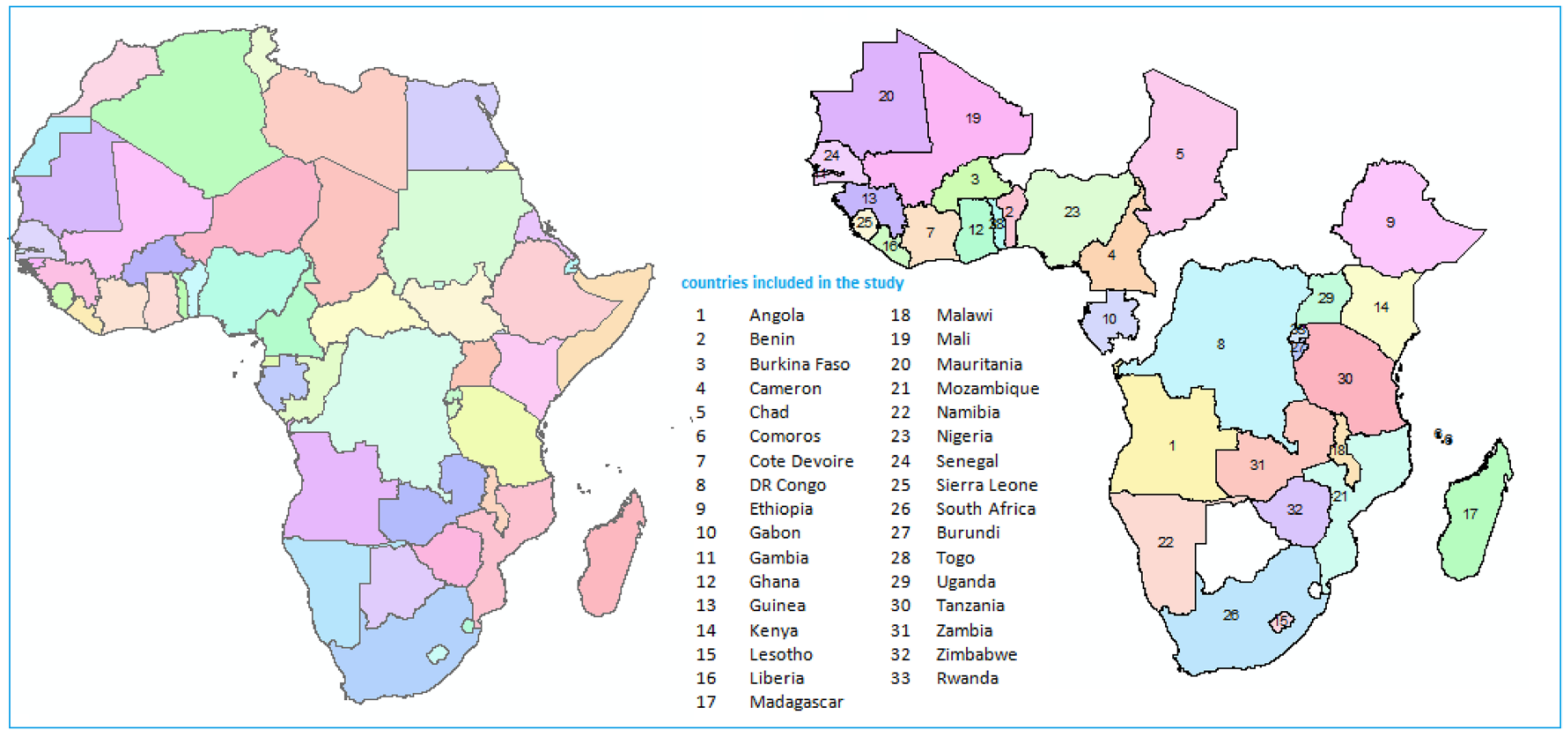
Eligible sub-Saharan African countries included in the study; we have created the map using ARC GIS.

The data for this study came from two sources: the Demographic and Health Survey (DHS), which is described in detail at https://dhsprogram.com. The data from 33 sSA countries (Fig. 1), including the global positioning systems (GPS) coordinates (latitude and longitude) of household clusters, were available (Table 1). In DHS, multistage sampling was used to select the sample for each survey in the countries included in this analysis. Hence, the first step of the sampling procedure involved the selection of clusters (enumeration areas (EAs)), followed by systematic household sampling within the selected EAs. The number of clusters is the first stage which is selected from the list of enumeration areas (EAs) created in the recent population census of each country and the households that are randomly selected in each of EAs. From the selected households, women aged 15–49 years are selected for an in-depth interview^21^. Moreover, the geographical covariates were extracted from the DHS site and were linked to the original individual DHS datasets through the cluster identifying number (ID). The key contextual climate factors in the study include the temperature, aridity index defined as the ratio of annual precipitation (0, most arid to 300, most wet), Daytime Land Surface Temperature (LST), and Enhanced Vegetation Index (EVI). The second data source is the National Aeronautics and Space Administration (NASA). From this source, the air pollution covariates such as global annual surface particulate matter (PM 2.5) concentration and the nitrogen dioxide (NO_2_) for 1998–2019 (v4.03) was estimated by the Atmospheric Composition Analysis Group. This data is available in the form of raster images (GeoTIFF) which are extracted using R software via the GPS locations (longitude and latitude). The data are publicly available at https://sedac.ciesin.columbia.edu/data/set/sdei-global-annual-gwr-pm2-5-modis-misr-seawifs-aod-v4-gl-03^22^. This dataset was combined with the original individual DHS datasets based on the community (enumeration areas) and the date of the survey. Air pollution covariates such as NO2 and PM2.5 for each of the EAs from 2012 to 2020 were obtained.

**Table 1:** Selection of study participants from 33 sSA countries with recent DHS reports from 2012 to 2022.

A total of 49 countries are located in Sub-Saharan Africa			
East African regions 18 countries	West African regions 17 countries	Central Africa regions 9 countries	Southern Africa regions 5 countries
A total of 16 countries were excluded for the following reasons			
6 countries were excluded ✓ 3 countries no DHS report ✓ 3 countries no GPS is available	4 countries were excluded ✓ 1 country with no DHS report ✓ 3 countries no GPS available	4 countries were excluded ✓ 1 country with no DHS report ✓ 3 countries no GPS available	2 Countries were excluded ✓ 2 countries where no GPS is available
A total of 33 countries included			
East African regions ✓ 12 countries (Burundi, Comoros, Ethiopia, Madagascar, Malawi, Mozambique, Rwanda, Tanzania, Uganda, Zambia, Zimbabwe, and Kenya) ✓ 7,595 PSU ✓ 124,106 U5C	West African regions ✓ 13 countries (Benin, Burkina Faso, Gambia, Ghana, Guinea, Ivery Coast, Liberia, Mali, Mauritania. Nigeria, Senegal, Sierra Leone, and Togo ✓ 6952 PSU ✓ 130,996 U5C	Central African regions ✓ 5 countries (Angola, Cameroon, Chad, Democratic Republic Congo, and Gabon) ✓ 2546 PSU ✓ 61,911U5C	Southern African regions ✓ 3 countries (South Africa, Lesotho, and Namibia) ✓ 1595 PSU ✓ 10,494 U5C
A total of ✓ 33 countries ✓ 18,688 PSU ✓ 327,507 U5C			

*PSU* Primary Sampling Unit, *U5C* under-five children, *DHS* Demographic and Health Survey Data, *sSA* sub-Saharan Africa.

### Variables

#### Outcome variables

To measure the symptoms of respiratory infections, mothers/caregivers were asked if each of their under-five children had experienced symptoms of ARI (Cough, short rapid breaths or difficulty breathing) and fever, each classified as binary outcome measures (yes, no), within 2 weeks before the DHS surveys. ARI was defined as a child who had a history of an illness in the 2 weeks preceding the survey with cough and breathing faster than usual with short, rapid breaths or had difficulty breathing^23^, and severe ARI (SARI) was defined as having all ARI with fever^24^.

#### Features (independent variables)

**Figure 2:**
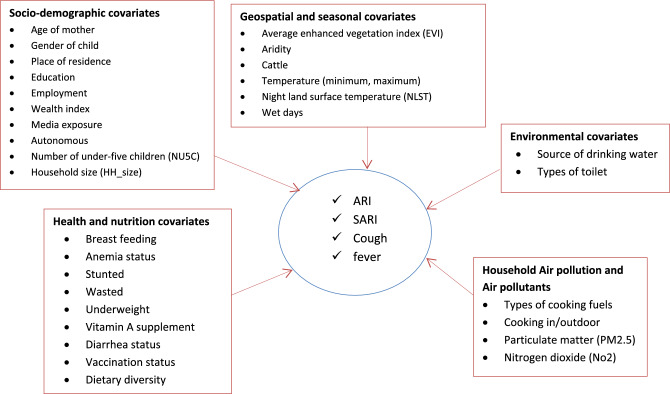
Conceptual framework for features description.

The independent variables extracted were based on a review of the literature^3,5–7,9,25,26^. The variables included in the analysis are summarized in the following framework (Fig. 2).

### Model building

#### Model building

Machine learning algorithms such as Logistic Regression (LR)^27^, Ridge regression^28^, Least Absolute Shrinkage and Selection Operator (LASSO) regression^29^, Elastic Net^30,31^, Decision trees^32^, K-Nearest Neighbors (KNN)^33^, Naïve Bayes^32,34,35^, Random Forest (RF)^31,36^, Bagged tree^37^, Boosting^37^ and Artificial Neural Network (ANN)^38,39^ were included in the analysis. All the statistical analyses were performed using the R software 4.3.1 for Windows (R Development Core Team). Moreover, the function *createDataPartition* in the R caret package splits the dataset using the stratified random sampling technique, which can minimize the bias of the data distribution and create balanced data.

#### Logistic regression (LR)

LR is a widely applied statistical model for binary classification problems. Let ${y}_{i}$ be the response variable for the ith child, assumed to follow the Bernoulli distribution and takes on the value 1 with a probability of ${\pi }_{i}=P({y}_{i}=1|{{\varvec{x}}}_{i})$, where ${{\varvec{x}}}_{i}={({x}_{1i}, . . . , {x}_{pi})}^{T}$ is the i^th^ child’s covariate vector, and value 0 with probability 1-${\pi }_{i}$. Then the logistic regression model with the logit link function can be given as:

1 $${\pi }_{i}=\frac{\text{exp}({\beta }_{0}+{{\varvec{x}}}_{i}^{T}{\varvec{\beta}})}{1+\text{exp}({\beta }_{0}+{{\varvec{x}}}_{i}^{T}{\varvec{\beta}})}.$$

where ${\beta }_{0}$ is the intercept term, and ${\varvec{\beta}}={({\beta }_{1}, . . . , {\beta }_{p})}^{T}$ is a p × 1 vector of estimated regression parameters on the logit scale. When we have many features (dimensionality), the traditional LR model has a few limitations: over-fitting, multicollinearity, and computational difficulties. To address these problems, we used regularization which is a GLM that imposes a penalty on the parameters to shrink them toward zero^27–31,40^.

*The ridge regression* (${L}_{2}$ regularization, which shrinks coefficients of correlated covariates towards each other) is obtained by maximizing the function with a penalized parameter $\lambda$ applied for all the parameters except the constant (intercept)^27,28^. The penalized likelihood formulation for ridge regression is given by (2)

2 $${l}_{\lambda }^{\text{R}}\left({\varvec{\beta}}\right)=\sum_{i=1}^{n}\left[{y}_{i}\left({{\varvec{x}}}_{i}^{T}{\varvec{\beta}}\right)-\text{log}\left(1+\text{exp}({{\varvec{x}}}_{i}^{T}{\varvec{\beta}}\right))\right]-\lambda \sum_{j=1}^{p}{{\varvec{\beta}}}_{j}^{2}$$

When the λ values are too large (λ → ∞), the coefficients of all the parameters tend to be zero, but when λ = 0, the ridge regression is equal to the traditional approach. The goal is to search for an optimal value between these two extremes.

*The LASSO regression* uses the ${L}_{1}$ penalty for variable selection and shrinkage. As such, if the $\lambda$ is large enough, it forces the coefficient to be zero which provides a lesser number of predictors^29^. The function for the lasso regression is given by **Eq. (3)

3 $${l}_{\lambda }^{\text{L}}\left({\varvec{\beta}}\right)=\sum_{i=1}^{n}\left[{y}_{i}\left({{\varvec{x}}}_{i}^{T}{\varvec{\beta}}\right)-\text{log}\left(1+\text{exp}({{\varvec{x}}}_{i}^{T}{\varvec{\beta}}\right))\right]-\lambda \sum_{j=1}^{p}\left|{{\varvec{\beta}}}_{j}\right|.$$

The optimal regularization parameter ($\lambda$) was determined using the nfold cross-validation techniques. The smaller the $\lambda$ value, the more the effect of regularization upon the number of covariates (features) in the model and their respective coefficients^31,41,42^. Thus, variables with non-zero estimates are considered important covariates for the outcome variable of interest.

*The elastic net regularization* is a combination of both **Eq. (2) and (3) penalties^30,31^. This method can effectively control for correlated features and also shrink the coefficients of non-informative features to zero^30,31,40,43^. The elastic net regression is given by (4)

4 $${l}_{\alpha }^{\text{El}}\left({\varvec{\beta}}\right)=\sum_{i=1}^{n}\left[{y}_{i}\left({{\varvec{x}}}_{i}^{T}{\varvec{\beta}}\right)-\text{log}\left(1+\text{exp}({{\varvec{x}}}_{i}^{T}{\varvec{\beta}}\right))\right]+\alpha \sum_{j=1}^{p}{{\varvec{\beta}}}_{j}^{2}+(1-\alpha )\sum_{j=1}^{p}\left|{{\varvec{\beta}}}_{j}\right|$$

All the GLM regularizations are operationalized in R programming software using the **glmnet** package^44^. In this paper, we trained the generalized linear model (GLM) estimators with common $\alpha$ values from the set {0, 0.5, 1}, where ($\alpha \hspace{0.17em}$= 0.0, 0.5 and 1.0 respectively refers to the ridge, elastic net and lasso penalty)^30,31,40^.

#### Random forest (RF)

RF is the popular supervised ML approach in applied statistics because of its applicability in both classification and regression^45–47^. It is also used for variable screening for dimension reduction^48–50^. It is a "tree-based" technique in which several decision trees are constructed from a random set of covariates and used to predict an outcome label for a subset of samples. It builds multiple trees (called the forest) and the decision is based on the majority votes over all the trees in the forest. This model is also used to select the important features^45–47,51^. The Gini Importance analysis was conducted through random forest ML approaches to identify the features that have the most impact on the likelihood of developing symptoms of respiratory infections among under-five children in sSA countries.

#### Naïve Bayesian (NB)

NB is a collection of ML classification algorithms built on Bayes theorem. These algorithms are built on two basic assumptions; the first is that every pair of features being classified is independent of others and hence “naïve”), and the second is that each makes an independent and equal contribution to the outcome^32,34,35^. For a binary outcome variable, a Bernoulli Naïve Bayesian algorithm is appropriate and given as

5 $$\text{P}\left(\text{y}|\text{x}\right)=\frac{\text{P}\left(\text{X}|\text{y}\right)p\left(y\right)}{P\left(X\right)}.$$

where X is the covariates and (X) is the predictors' prior probability, P(y) is referred to as the probability before evidence is seen or the prior. P(X|y) is known as the likelihood.

#### Decision trees (DT)

The given dataset is repeatedly split into increasingly similar groups based on the variable that maximizes the similarity of resulting groups^32^. The nodes of the DT normally have multiple levels where the topmost or first node is known as the root node. The predictions and classifications are made by evaluating the new individual according to the established criteria. The DT classifier was constructed using the R package rpart, and the classification and regression tree (CART) was applied to build binary trees.

**Figure 3:**
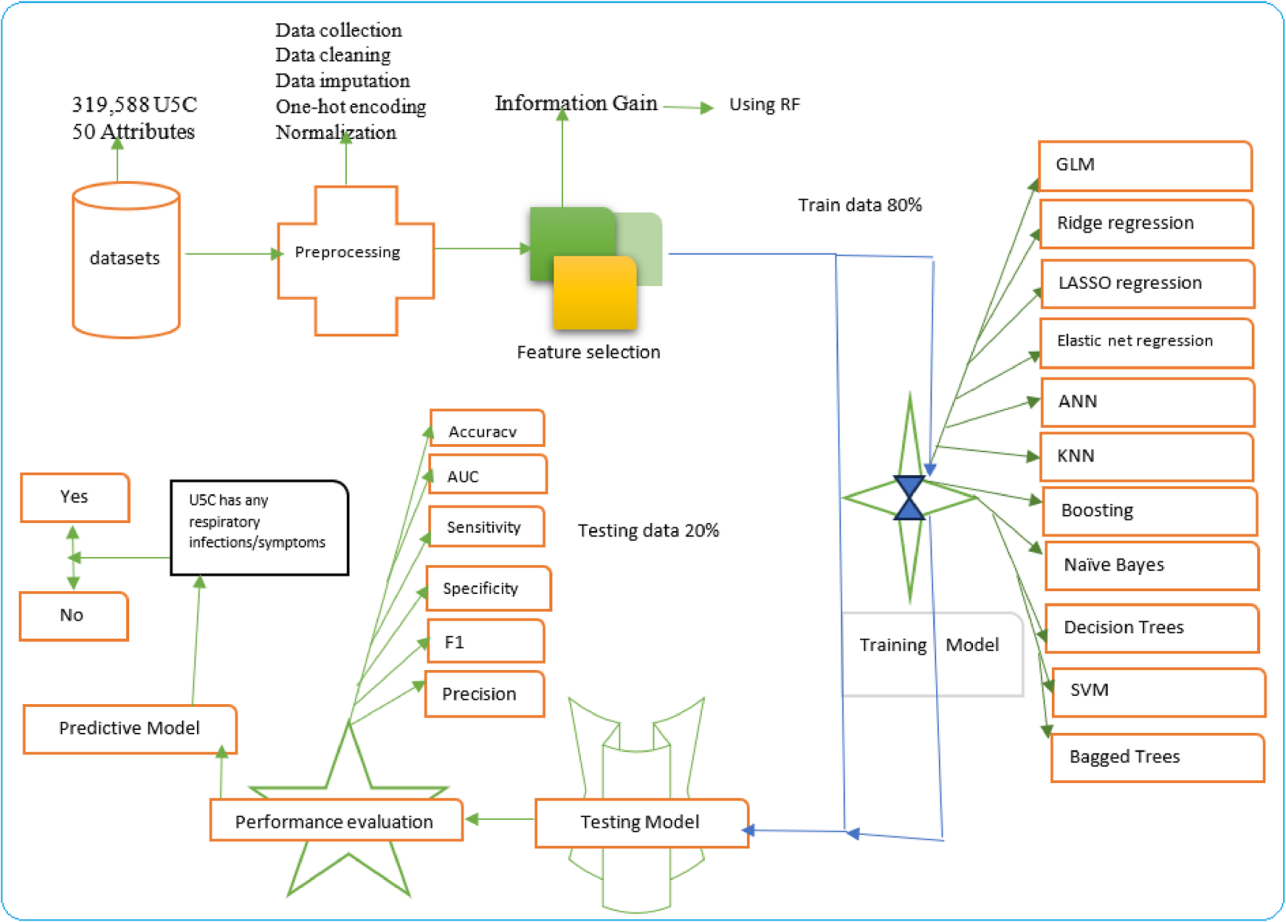
Overview flow chart of the machine learning algorithms used for predicting U5C respiratory infections/symptoms.

Figure 3 below shows the research workflow. Before performing any statistical analysis, the data were pre-processed, which was followed by feature selection. The data management, including missing values, the existence of outliers, and illogical values was checked. The missing value imputation process was carried out iteratively until 100% completeness of all variables was achieved. Specifically, we checked the missing values in the dataset. A value was excluded from the analysis if missing-ness was less than 10% for any variable including the study. However, mean imputation for continuous variable and mode imputation methods for categorical data were used to fill in the missing values if it is greater than 10%. The three-step approach consisted of feature selection, model comparison, and selection of the best ML models and interpretation. The random forest, which is one of the common approaches to identifying important features^46,47,50–52^, was used. It generates 1000 trees and selects the Gini criteria to compute the importance of each feature, the second quartile (median) was considered as a cut of point for selecting important features. Only the symptom of ARIs, as an outcome (dependent (target)) variable for the machine learning parts, was used. To assess the performance of the given ML classifications, we randomly split the dataset into two: training (80%) and (20%) testing datasets. The performances of the given ML models are evaluated using sensitivity, specificity, the area under the curve, and accuracy^31,41,42,53–56^ which are calculated using the observed data as the gold standard.

After constructing the ML models, sensitivity, specificity, accuracy, and area under the curve (AUC) were calculated to test the performance. The AUC gives an aggregated value which explains the probability that a random sample would be correctly classified by each of the ML algorithms^54,57^. The AUC of the receiver characteristics curve (ROC) averaged over 10 cross-validation folds (ten repeats)^54^, which partitions the original sample into ten disjoint subsets, uses nine of those subsets in the training process, and then makes predictions about the remaining subset. When viewing the area under the receiver operating curve (AUC-ROC), the classifiers that provide curves closer to the top-left corner represent a reliable performance and hence the RF model is more accurate in distinguishing the diagnosis of symptoms of respiratory infections among children under 5 years. The ROC curve is a virtual demonstration used to explain the diagnostic capability of binary classifiers which is a plot of the specificity (1-false positive rate (FPR)) on the horizontal axis and sensitivity-true positive rate (TPR) on the vertical axis. Then the identified best-fit model is used to predict the respiratory symptoms in another dataset, known as the test dataset^31,41,42,53–55^.

### Compliance with ethics guidelines

The protocol for the sub-Saharan DHS was approved by the Humanities and Social Sciences Research Ethics Committee (HSSREC/00005776/2023) of the University of KwaZulu-Natal. The authors obtained permission from the demographic and health survey (DHS) program to download and use the data for this analysis and the need for informed consent was waived.

## Results

**Figure 4:**
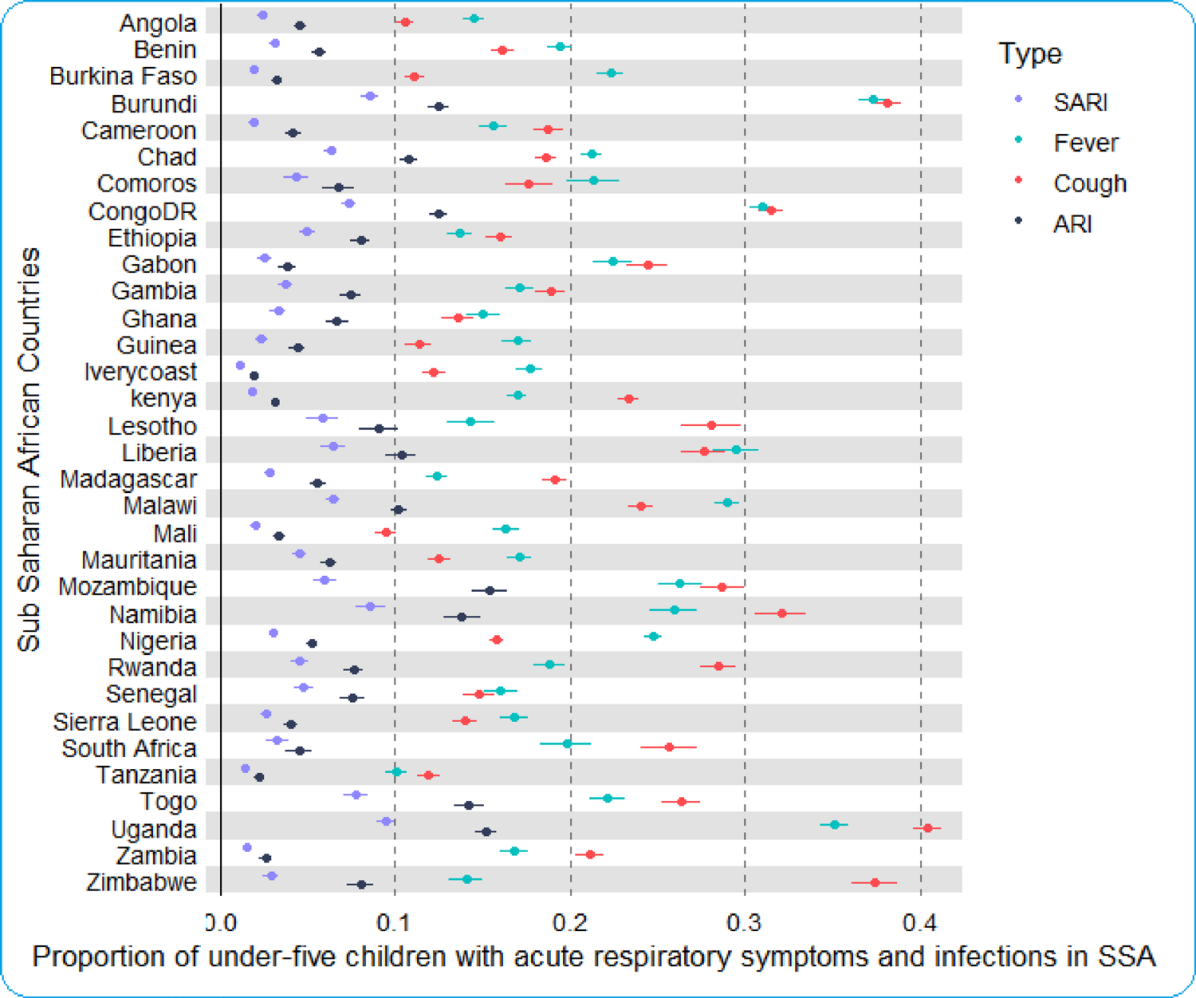
Proportion of under-five children with different AR infections and symptoms across sSA countries.

Table 1 presents the prevalence of symptoms of respiratory infections among under-five children from 33 sSA countries. A total of 327,507 under-five children were included in the study. The overall prevalence of symptoms of ARI, SARI, cough, and fever for all countries was 7.10, 4.19, 20.61, and 21.02% respectively. However, there are inequalities in the symptoms of respiratory infections among under-five children across sSA countries (Table 1, Fig. 4).

**Table:** 

The number of under-five children across the DHS waves for each country and the prevalence of symptoms of respiratory infections among U5C children in sSA							
Survey countries	Survey year	Weighted sample	Percent	Children with symptoms of			
				ARI n (%)	SARI n (%)	Cough n (%)	Fever n (%)
Angola	2015	13,439	4.10	606 (4.51)	317 (2.36)	1416 (10.54)	1934 (14.39)
Benin	2017	12,529	3.83	702 (5.60)	395 (3.15)	2016 (16.09)	2427 (19.37)
Burkina Faso	2021	11,763	3.59	377 (3.20)	230 (1.96)	1308 (11.12)	2622 (22.29)
Burundi	2016	12,432	3.80	1549 (12.46)	1063 (8.55)	4740 (38.13)	4639 (37.31)
Cameroon	2017	8986	2.74	373 (4.15)	167 (1.86)	1687 (18.77)	1387 (15.44)
Chad	2015	16,644	5.08	1794 (10.78)	1053 (6.33)	3092 (18.58)	3531 (21.21)
Comoros	2011	2916	0.89	200 (6.86)	130 (4.46)	516 (17.70)	622 (21.33)
Congo democratic	2013	16,960	5.18	2098 (12.37)	1244 (7.33)	5306 (31.29)	5229 (30.83)
Ivory Coast	2017	9888	3.02	188 (1.90)	111 (1.12)	1187 (12.00)	1724 (17.44)
Ethiopia	2016	9911	3.03	795 (8.02)	493 (4.97)	1583 (15.97)	1354 (13.66)
Gabon	2019	5882	1.80	233 (3.96)	150 (2.55)	1426 (24.24)	1311 (22.29)
Gambia	2019	7764	2.37	578 (7.44)	288 (3.71)	1463 (18.84)	1324 (17.05)
Ghana	2014	5544	1.69	364 (6.57)	178 (3.21)	744 (13.42)	821 (14.81)
Guinea	2018	6633	2.03	287 (4.33)	157 (2.37)	744 (11.22)	1123 (16.93)
Kenya	2022	18,705	5.71	582 (3.11)	340 (1.82)	4328 (23.14)	3143 (16.80)
Lesotho	2014	2818	0.86	259 (9.19)	167 (5.93)	789 (28.00)	405 (14.37)
Liberia	2019	4083	1.55	518 (10.19)	325 (6.39)	1379 (27.13)	1471 (28.94)
Madagascar	2021	11,647	3.56	651 (5.59)	323 (2.77)	2217 (19.03)	1438 (12.35)
Malawi	2015	16,209	4.95	1648 (10.17)	1044 (6.44)	3889 (23.99)	4687 (28.92)
Mali	2018	9175	2.80	311 (3.39)	189 (2.06)	866 (9.44)	1497 (16.32)
Mauritania	2019	10,956	3.35	672 (6.13)	495 (4.52)	1372 (12.52)	1874 (17.10)
Mozambique	2015	4954	1.51	758 (15.30)	295 (5.95)	1415 (28.56)	1300 (26.24)
Namibia	2013	4426	1.35	604 (13.65)	380 (8.59)	1381 (31.20)	1128 (25.49)
Nigeria	2018	30,597	9.34	1603 (5.24)	940 (3.07)	4816 (15.74)	7535 (24.63)
Rwanda	2019	7758	2.37	587 (7.57)	351 (4.52)	2208 (28.46)	1468 (18.92)
Senegal	2019	5726	1.75	430 (7.51)	270 (4.72)	848 (14.81)	920 (16.07)
Sierra Leone	2019	8878	2.71	354 (3.99)	233 (2.62)	1231 (13.87)	1473 (16.59)
South Africa	2016	3250	0.99	150 (4.62)	108 (3.32)	820 (25.23)	647 (19.91)
Tanzania	2022	10,197	3.11	221 (2.17)	145 (1.42)	1197 (11.74)	1011 (9.91)
Togo	2013	6460	1.97	922 (14.27)	498 (7.71)	1698 (26.28)	1413 (21.87)
Uganda	2016	14,378	4.39	2164 (15.05)	1349 (9.38)	5766 (40.10)	5027 (34.96)
Zambia	2019	9308	2.84	241 (2.59)	142 (1.53)	1948 (20.93)	1549 (16.64)
Zimbabwe	2015	53,691	1.74	445 (7.82)	166 (2.92)	2103 (36.95)	796(13.99)
Total		327,507	100	23,264 (7.10)	13,736 (4.19)	67,499 (20.61)	68,830 (21.02)

The preliminary analysis for symptoms of ARI using a generalized linear model (logistic regression) with the type of features and their relative importance values separately reported for socio-demographic, geospatial, health and nutrition, and environmental covariates are summarized in Table 2. The results of the variables showed that among the socio-demographic variables: age of mother, place of residence, and media exposure, from health nutrition-related features: breast-feeding, nutrition status (stunting, wasting, and underweight), and dietary diversity, from geospatial covariates: enhanced vegetation index, aridity, wet day, and the minimum temperature were positive predictors of the symptoms of ARIs. Additionally, environmental features: source of drinking water and toilet facility; air pollution features: fuel type, cooking place, PM2.5, and spatial locations (longitude, latitude) statistically and significantly affected the symptoms of ARI among under-five children in sSA countries (Table 2).

**Table 2:** Preliminary analysis of the effects of different variables on the outcome variables and the relative importance of each of the features on the target variable.

Characteristic	Feature name	Category	Cough		Fever		ARI		SARI	
			OR (95% CI)	Relative importance (%)	OR (95% CI)	Relative importance (%)	OR (95% CI)	Relative importance (%)	OR (95% CI)	Relative importance (%)
Socio-demographic features	Age of mother		0.98 (0.97,0.99)***	97.15	0.98 (0.96,0.99)***	69.85	0.94 (0.92,0.96)***	97.66	0.97 (0.94,1.00)	100
	Gender	Male	0.99 (0.972,1.01)	24.42	0.98 (0.91,1.06)	18.45	1.01 (0.98,1.04)	23.27	1.02 (0.98,1.53)	24.79
		Female	1		1		1		1	
	Place residence	Rural	0.99 (0.96,1.01)	12.69	1.11 (1.08,1.14)***	13.55	1.07 (1.03,1.11)**	10.69	1.19 (1.03,1.18)***	11.23
		Urban	1		1		1		1	
	Education mother	Literate	0.84 (0.82,0.86)***	14.27	0.98 (0.96,1.02)	11.29	0.96 (0.92,0.99)*	13.09	0.97 (0.93,1.34)	15.44
		Illiterate	1		1		1		1	
	Education father	Literate	0.87 (0.85,0.89)***	12.12	0.96 (0.94,0.98)**	8.75	0.87 (0.84,0.90)***	11.49	0.89 (0.87,0.93)***	11.53
		Illiterate	1		1		1		1	
	Employment mother	Employed	0.81 (0.79,0.83)***	22.49	0.79 (0.77,0.80)***	12.49	0.82 (0.79,0.85)***	15.96	0.79 (0.75,0.85)***	14.88
		Unemployed	1		1		1		1	
	Employment father	Employed	0.86 (0.82,0.90)***	5.95	0.87 (0.83,0.91)***	4.75	0.79 (0.74,0.85)***	6.14	0.86 (0.74,0.94)***	6.37
		Unemployed	1		1		1		1	
	Wealth index	Poor	1.04 (1.02,1.06)***	18.95	1.17 (1.14,1.19)***	19.81	1.02 (0.97,1.04)	18.08	1.09 (1.05,1.15)***	20.88
		Non-poor	1		1		1		1	
	Media exposure	Yes	0.89 (0.84, 0.88)***	26.39	0.98 (0.96, 0.99)**	15.38	0.98 (0.95, 1.01)	18.53	0.98 (0.95,1.02)	21.07
		No	1		1		1		1	
	Marital status	Married	0.98 (0.950,1.01)	16.17	0.98 (0.96,1.02)	12.17	1.02 (0.96,1.07)	15.18	0.98 (0.93,1.04)	17.54
		Not married	1		1		1		1	
	Size of child	Small	1.14 (1.12,1.17)***	20.6	1.10 (1.08,1.13)***	16.67	1.18 (1.14,1.23)***	20.17	1.19 (1.14,1.24)***	21.49
		Average +	1		1		1		1	
	Place delivery	Home	0.91 (0.89,0.93)***	19.47	0.94 (0.922,0.96)***	16.32	0.99 (0.96,1.03)	20.6	0.98 (0.97,1.02)	21.31
		Health facility	1		1		1		1	
	Contraceptive use	Yes	1.17 (1.15.201)***	22.43	1.10 (1.06,1.12)***	16.48	1.11 (1.07,1.15)***	21.6	1.11 1.07,1.16)***	22.6
		No	1		1		1		1	
	HH_size	< 2	0.80 (0.79, 0.82)***	14.64	0.97 (0.95,0.99)***	16.99	0.92 (0.89, 0.96)***	20.86	0.93 (0.89, 0.98)*	22.07
		> = 3	1		1		1		1	
	NU5C	02-Jan	0.91 (0.89,0.93)***	1.99	0.92 (0.90,0.94)***	17.41	0.95 (0.92,0.98)***	22.23	0.94 (0.89,0.98)**	24.43
		> = 3	1		1		1		1	
Health and nutrition related features	Breastfeeding	Yes	0.96 (0.94, 0.97)***	22.11	0.99 (0.97, 1.01)	9.82	0.82 (0.80, 0.84)***	10.92	0.85 (0.82, 0.88,)**	13.2
		No	1		1		1		1	
	Anemia status	Yes	1.07 (1.05, 1.09)**	22.26	1.02 (0.99,1.04)	2.33	1.08 (1.05,1.11)***	3.56	1.05 (1.01,1.09)**	5.83
		No	1		1		1		1	
	Stunting	Yes	1.02 (0.99,0.1.04)	12.14	1.09 (1.07, 1.12)***	5.37	1.02 (0.98, 1.05)	7.97	1.07 (1.02, 1.12)*	9.67
		No	1		1		1		1	
	Wasting	Yes	1.07 (1.02,1.13)**	2.67	1.19 (1.13,1.25)***	22.88	1.12 (1.04,1.21)**	25.22	1.18 (1.08,1.29)***	26.13
		No	1		1		1		1	
	Underweight	Yes	1.07 (1.03,1.12)***	6.44	1.12 (1.08,1.16)***	13.27	1.12 (1.05,1.18)***	18.38	1.17 (1.09,1.26)***	19.97
		No	1		1		1		1	
	Vitamin A sup	Yes	0.72 (0.71, 0.73)***	35.32	0.78 (0.77,0.79)***	5.11	0.77 (0.75, 0.79)***	5.64	0.72 (0.69,0.74)***	5.6
		No	1		1		1		1	
	Vaccination	Yes	0.89 (0.88, 0.92)***	17.29	0.84 (0.82,0.86)***	17.43	0.77 (0.75, 0.80)***	21.6	0.75 (0.72,0.78)***	22.98
		No	1		1		1		1	
	Dietary diversity	< minimum	1.13 (1.10,1.16)***	22.21	1.19 (1.16,1.21)***	71.83	1.10 (1.07,1.14)***	60.9	1.08 (1.03,1.13)**	74.07
		Minimum	1		1		1		1	
	Diarrhea status	Yes	2.71 (2.66,2.77)***	59.54	4.07 (3.99,4.16)***	63.74	2.77 (2.69,2.86)***	70.48	3.67 (3.54,3.81)***	69.19
		No	1		1		1		1	
Geospatial features	EVI		1.00 (0.99, 1.01)	95.15	0.97 (0.96,0.98)***	77.21	0.99 (0.97,1.01)	93.49	0.98 (0.96, 0.99)**	93.87
	Aridity		1.00 (0.98,1.02)	69.22	1.12 (1.10,1.14)***	68.75	1.00 (0.98,1.01)	87.47	1.03 (0.99, 1.07)	85.58
	LST		1.01 (1.00,1.02)	94.97	1.02 (1.01,1.03)**	83.25	1.02 (1.01,1.04)**	64.57	1.05 (1.02, 1.07)**	63.95
	Cattle		1.04 (1.03,1.05)***	94.62	1.00 (1.00,1.01)	65.12	1.04 (1.02,1.05)**	63.16	1.01 (0.99, 1.03)	60.66
	MaxT		0.92 (0.91,0.93)***	66.55	1.05 (1.04,1.07)***	66.65	0.95 (0.93,0.96)**	66.77	0.98 (0.95,1.00)	65.9
	MinT		1.02 (1.1.01,1.03)***	66.14	1.00 (0.99,1.02)	12.45	1.02 (1.00,1.04)	16.19	1.01 (0.99,1.03)	16.61
	Wetday		1.07 (1.05,1.09)***	69.74	0.94 (0.93,0.96)***	10.71	1.08 (1.05,1.11)***	13.34	1.02 (1.00,1.05)**	14.99
Environmental features	Water	Improved	0.98 (0.96, 0.99**	16.92	0.95 (0.93, 0.96)***	1.95	0.93 (0.89, 0.96)***	1.79	0.94 (0.90, 0.98)	2.39
		Unimproved	1		1		1		1	
	Toilet facility	Improved	0.84 (0.82,0.85)***	14.26	0.92 (0.90,0.94)***	11.26	0.91 (0.88,0.94)***	13.55	0.89 (0.86,0.93)***	14.43
		Unimproved	1		1		1		1	
Household air pollution and air pollutants	Cooking fuels	Cleaned	0.97 (0.94,1.03)	0	0.93 (0.89,0.96)***	0	0.82 (0.78,0.87)***	0	0.88 (0.82,0.95)**	0
		Uncleaned	1		1		1		1	
	Cooking place	indoor	1.11 (1.09, 1.13) ***	20.47	1.03 (1.01,1.05)***	16.56	1.04 (1.01,1.07)**	20.29	1.02 (0.99,1.06)	21.38
		outdoor	1		1		1		1	
	PM2.5		1.00 (0.99,1.01)	93.15	1.03 (1.02,1.04)***	98.79	1.01 (1.00,1.03)	100	0.92 (0.90,1.04)	93.65
	No2		1.03 (1.02, 1.04)***	93.1	0.93 (0.92,0.94)***	71.27	1.03 (1.02,1.04)**	88.02	0.92 (0.90,0.94)**	87.25
	Longitude		1.01 (1.01,1.03)***	99.31	1.07 (1.06,1.08)***	100	1.05 (1.05,1.08)***	94.49	1.11 (1.09,1.13)***	94.84
	Latitude		1.02 (1.01,1.04)***	100	1.06 (1.05,1.07)***	83.37	1.04 (1.02,1.05)***	94.67	1.06 (1.04,1.09)***	93

**Figure 5:**
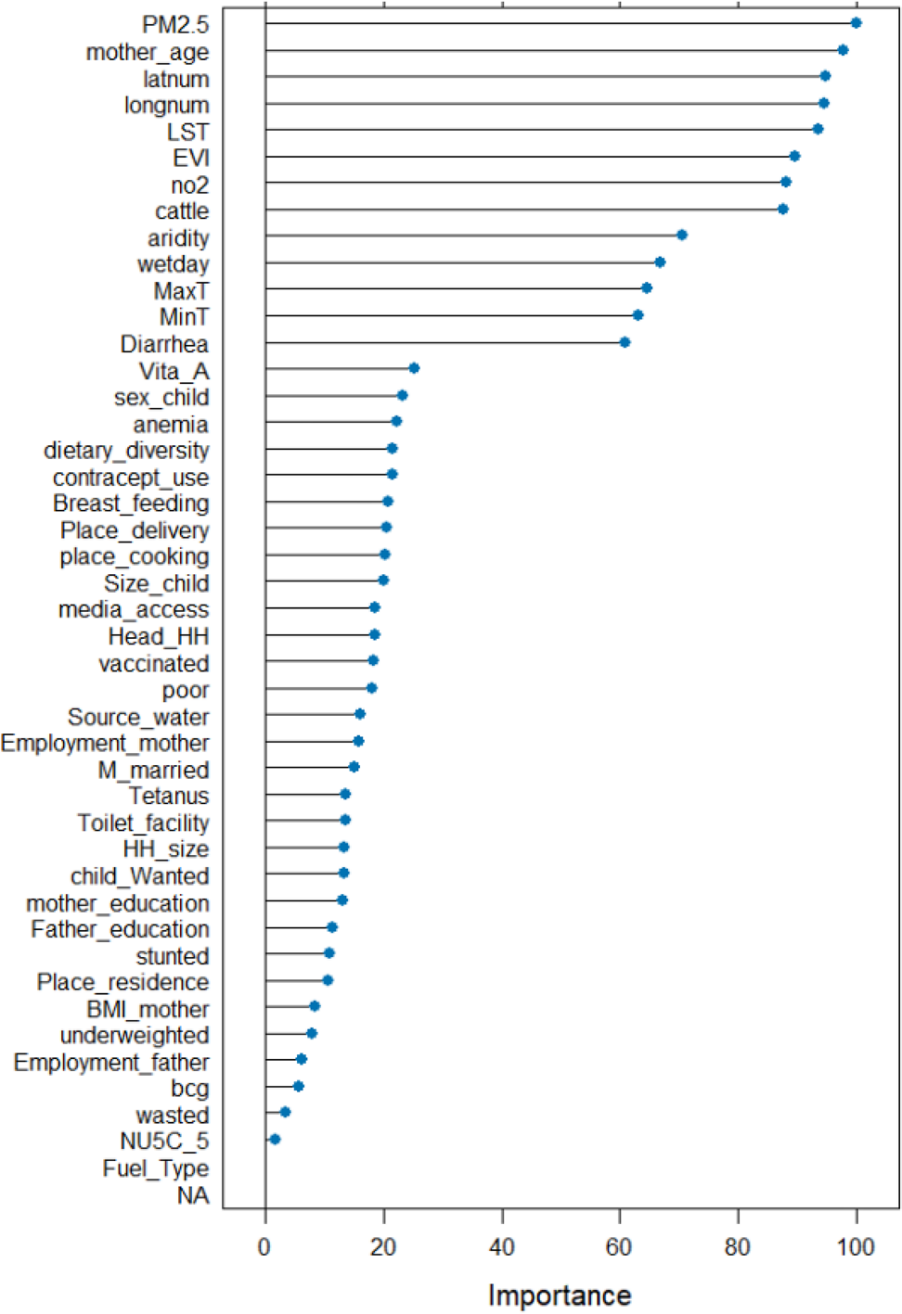
Feature importance scores based on random forest approach.

The relative importance results in a features score larger than the second quartile (20.3) was considered as a cut-off point for selecting important features and these were used for the subsequent machine learning models. As a result, 21 features are retained for the subsequent analysis. As shown in Fig. 5, the top features with strong influences on the symptoms of ARI among under-five children in sSA countries were air pollutants and climatic factors: household air pollution and air pollutants such as particulate matter (PM2.5), cooking indoors and outdoors, nitrogen dioxide and types of fuel. The features from geospatial/climate variables; spatial location (longitude, latitude), LST, EVI, Cattle, maximum/minimum temperature, aridity, and wet days have a relative importance score greater than the second quartile (20.3%). Whereas only the mother's age and sex of a child from socio-demographic and diarrhea status and vitamin A supplement from health-related features were selected for further ML models to predict the symptoms of ARIs among under-five children across sSA countries. Finally, the proposed ML models such as GLM (logistic regression), Ridge, LASSO, Elastic net, ANN, KNN, Boosting, Naïve Bayes, DT, RF, and Bagged Trees were employed based on the selected features to classify the diagnosis of symptoms of ARIs of the under-five children in sSA countries (Fig. 5).

The model evaluation and accuracy scores of different supervised machine learning models were done by randomly sampling 20% of the dataset as a test sample (Table 3). Table 3 revealed that there is no substantial difference in accuracies of the different MLAs that can predict the symptoms of ARI among under-five children in sSA countries. The highest model performance was obtained by Random Forest, Boosting, ANN, and Bagged trees with AUCs of 0.77, 0.76, 0.74, and 0.74 respectively. The lowest model performance was observed for DT and NB with AUC = 0.68 and 0.70 respectively (Table 3, Supplementary Fig. S1).

**Table 3:** The performance of the prediction models based on different classifications using a test dataset with 95% CI.

Algorithms	Sensitivity (95% CI)	Specificity (95% CI)	AUC (95% CI)	Accuracy (95% CI)
GLM	0.64 (0.63, 0.66)	0.67 (0.64, 0.69)	0.72 (0.70, 0.73)	0.65 (0.64, 0.67)
Ridge	0.89 (0.88, 0.90)	0.36 (0.34, 0.39)	0.71 (0.70, 0.73)	0.71 (0.70, 0.73)
Lasso	0.89 (0.88, 0.90)	0.37 (0.34, 0.39)	0.72 (70, 0.73)	0.71 (0.69, 0.72)
elastic-net	0.89 (0.88, 0.90)	0.36 (0.34, 0.39)	0.72 (0.70, 0.73)	0.70 (0.69, 0.72)
ANN	0.64 (0.63, 0.66)	0.71 (0.68, 0.73)	0.74 (0.73, 0.75)	0.67 (0.65, 0.68)
KNN	0.84 (0.83, 0.86)	0.43 (0.40, 0.45)	0.71 (70, 0.73)	0.70 (0.69, 0.72)
NB	0.59 (0.57, 0.61)	0.72 (0.69, 0.74)	0.70 (0.68, 0.71)	0.63 (0.61, 0.65)
Bagged tree	0.80 (0.78, 0.81)	0.53 (0.50, 0.56)	0.74 (0.72, 0.75)	0.71 (0.69, 0.72)
**RF**	**0.81 (0.80, 0.83)**	**0.55 (0.52, 0.58)**	**0.77 (0.75, 0.78)**	**0.72 (0.71, 0.73)**
Boosting	0.82 (0.81, 0.84)	0.53 (0.50, 0.55)	0.76 (0.74, 0.77)	0.72 (0.71, 0.74)
DT	0.86 (0.85, 0.88)	0.40 (0.37, 0.73)	0.68 (66, 0.70)	0.71 (0.69, 0.72)

*GLM* generalized linear model, *ANN* artificial neural network, KNN K nearest neighbor, *NB* Naïve Bayes, *RF* Random Forest, *DT* decision tree, *AUC* area under curve, *CI* confidence interval.

The selected machine learning algorithm in bold.

## Discussion

This study explores a full statistical analysis of covariates associated with the ARIs among under-five children in sub-Saharan African countries, employing both descriptive data exploration and advanced machine learning algorithms. This study highlights a large variation in country-level prevalence of symptoms of ARIs among under-five children. Previous literature revealed that the distribution of the prevalence of ARIs varies from country to country^6–8,58^ and from district to district within the same country^7,58–60^.

One of the aims of this study was to apply ML algorithms to identify the key determinants (features) of ARIs among under-five children using a large dataset across sub-Saharan African countries. This is the first study to demonstrate the implementation of ML algorithms for predicting acute respiratory infection rates in sSA countries. The result of this study showcases the superior predictive capability powers of the MLA as compared to other conventional statistical techniques in identifying features linked to ARIs. The result is not surprising since MLA has been revealed to outperform traditional statistical models in several fields of the machine^61–64^. We have employed several ML techniques, to assess their predictive power capabilities. Evaluating the performance of these ML techniques, we investigated that all the techniques employed in this study achieved ROC values above the optimal threshold value (0.5). Using novel machine learning algorithms (MLA), our analysis of the multi-country DHS datasets strongly indicated the association of air pollution and environmental variables with the symptoms of ARI among children in sSA counties. In our study, PM2.5 was the most influential variable increasing the risk of ARI, together with NO_2_. Both PM2.5 and NO_2_ have been associated with the occurrence of respiratory infections^11,12,16,65^. Specifically, the support vector machine algorithm^66,67^ has previously shown that ARI is associated with NO_2_. Those previous researchers applied parametric linear models and semi-parametric and generalized additive models^68–71^ to determine the effects of air pollutants on symptoms of respiratory infections. To the best of our knowledge, few studies are using machine learning models to determine the association between air pollutants and human health^72–75^, and none have used ML models to determine the effects of air pollutants on children's symptoms of respiratory infections across the sub-Saharan regions. In this study, climate factors, such as temperature, wet day, and spatial location (longitude, latitude), were among the top features associated with the symptoms of respiratory infections. This is consistent with the previous studies^76–79^ that the temperature affects the occurrence of the symptoms of ARIs.

Nowadays, with the availability of large health-related data repositories (such as electronic medical records) and advances in computing power, classical statistical analysis is being combined with advanced machine learning algorithms to predict and classify the target variables (outcomes)^80–82^. The feature selection and feature relevance become prominent, especially in datasets with many features (independent variables)^37,52,81–83^. The RF approach has been also used for feature selection in previous studies^46,47,52,74^. Using this approach, we found that the most important features are particulate matter, age of the mother, spatial location (longitude, latitude), land surface temperature, enhanced vegetation index, nitrogen dioxide, aridity, wet day, temperature, and others were identified, and the similar result was obtained from previous studies^6–8,58,84–86^. In the study, all the ML classification approaches achieved greater accuracy in predicting/diagnostics of symptoms of ARI over traditional models like GLM also in line with studies on target variables^46,47,52,74,75,87^ elsewhere. The study used large nationally representative datasets of 33 sSA countries in examining and selecting the important features to diagnose the symptoms of ARIs. Again, this large dataset made it possible to apply the high-level ML approaches that confirm the accuracy of the findings. However, this study has some limitations. Firstly, we considered only one recent DHS dataset for each country, and hence we did not model the variables over time. Secondly, the data is cross-sectional so we can only make conclusions on statistical association (not causality). Thirdly, the study (survey) is conducted in different survey years and the comparison made on prevalence by country may mislead the readers. Lastly, even though the random forest machine learning method is commonly used for feature selection, other methods may prioritize features differently. Therefore, our future focus will be to include the temporal effects to draw inferences over time and possibly causality.

## Conclusion

The present study tried to assess the performance of various supervised machine-learning algorithms for the prediction of symptoms of respiratory infections using data from DHS and NASA sources. In this study, before we started the feature selection process, our dataset contained a total of 51 features and 327,507 under-five children. Feature selection is essential for the classification and prediction of certain target variables. Using the random forest approach, the ranking of the contributions of the features was determined by using the average Gini Importance method and only 21 features were retained for further ML models. It was found that particulate matter (PM2.5), age of the mother, spatial location (longitude, latitude), land surface temperature, enhanced vegetation index, nitrogen dioxide, aridity, wet day, and temperature are the most important predictors of symptoms of ARI among children in sSA countries. Those selected features have scores greater than the second quartile (median), which is used as a rule of thumb for dimension reduction of features. The present study attempted to identify the best ML algorithms for the prediction of symptoms of ARI using nationwide cross-sectional data from 33 SSA countries. The performances of these ML models were compared using different statistical merits such as sensitivity, specificity, accuracy, and AUC. Air pollution is a leading cause of symptoms of respiratory infections (fever, cough, ARI, and SARI) among children and adults. In addition, the ML algorithms are more accurate for the prediction of the symptoms and this result may apply to other target variables, for large data sets. The findings of this study established the potential of the ML techniques in predicting the presence of ARI among under-five children across sSA countries. This opens up the opportunities for development of automated screening tools and decision support systems which may assist the concerned bodies in diagnosing and managing the ARIs among under-five children in the region. Moreover, the spatial location (longitude, latitude) is one of the influential features in predicting and diagnostic symptoms of ARIs, hence if the spatial model is integrated with the ML models, it is possible to identify and flag under five children who are at most risk, such that data-driven intervention can be targeted to communities where those children live.

### Supplementary Information

Supplementary Figure S1.

## Data Availability

The datasets generated and analyzed during the current study are available subject to permission from the DHS program, in the DHS repository (https://dhsprogram.com/data).
