# Supplementary material for: Factors of acute respiratory infection among under-five children across sub-Saharan African countries using machine learning approaches
Source: Sci Rep. 2024 Jul 9;14:15801. doi: 10.1038/s41598-024-65620-1 (PMC11233665; doi:10.1038/s41598-024-65620-1)
Supplement: Supplementary file 1 — Supplementary Figure S1. [file 41598_2024_65620_MOESM1_ESM.pdf]

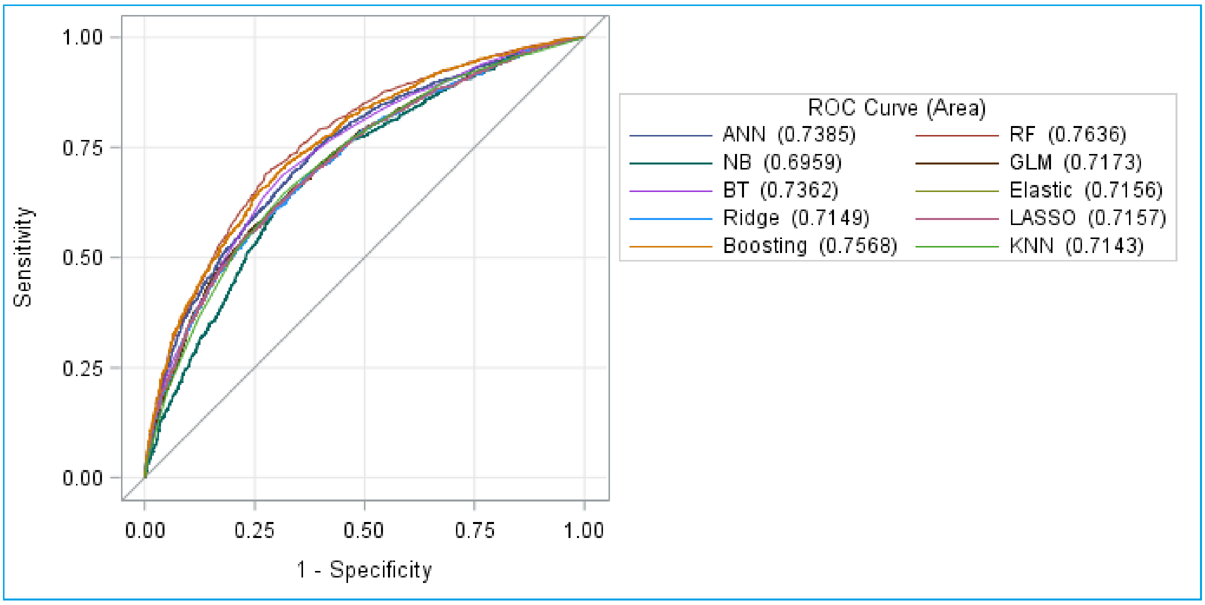

Supplementary Figure. S1: ROC curves for different machine learning models in predicting symptoms of respiratory infections among under-five children in sub-Saharan African countries: the result is based on the test dataset.
